# Anti-PLA2R antibody levels and clinical risk factors for predicting relapse in primary membranous nephropathy

**DOI:** 10.3389/fimmu.2026.1780071

**Published:** 2026-09-25

**Authors:** Hongju Wang, Huijuan Ma, Huijing Wang

**Affiliations:** 1 Department of Nephrology, Jining No.1 People’s Hospital, Jining, China; 2 Rheumatology, Renji Hospital, Shanghai Jiao Tong University School of Medicine, Shanghai, China

**Keywords:** anti-PLA2R antibody, primary membranous nephropathy, prognosis, relapse, risk factor

## Abstract

**Objectives:**

To explore the predictive value of anti-phospholipase A2 receptor (PLA2R) antibody levels and clinical risk factors for relapse in patients with primary membranous nephropathy (pMN) who achieved remission after immunosuppressive therapy.

**Methods:**

This study was a single-center, retrospective, cohort study. Patients with biopsy-proven pMN from Jining No.1 People’s Hospital, who had a follow-up period of at least 12 months, were enrolled. Three landmark time points were evaluated: baseline and 3 and 6 months after treatment initiation. Multivariable Cox regression, receiver operating characteristic curve analysis, and Kaplan–Meier survival analysis were performed. Cox model discrimination and fit were assessed using Harrell’s C-index and the Akaike information criterion (AIC), respectively.

**Results:**

A total of 154 patients with PLA2R-associated pMN were included in the study. Among the 96 patients (62.34%) who achieved remission (either partial or complete) within 12 months, 17 (17.71%) experienced a relapse; the median time from treatment initiation to relapse was 23 months. The baseline anti-PLA2R antibody level in the relapse group was significantly higher than that in the non-relapse group (*p* < 0.001). In the 3-month landmark model, anti-PLA2R antibody seropositivity at 3 months (≥ 14 RU/mL) was independently associated with a higher subsequent risk of relapse (HR 9.44, 95% CI 2.69-33.21; p < 0.001), whereas a greater increase in serum albumin from baseline to 3 months was associated with a lower relapse risk (HR 0.85, 95% CI 0.74-0.98; p = 0.03). The 3-month landmark model demonstrated better model fit and discrimination than the 6-month landmark model, with a lower AIC (123.41 vs. 138.84) and a higher C-index (0.83 vs. 0.66).

**Conclusion:**

In this exploratory study, anti-PLA2R antibody seropositivity and a blunted increase in serum albumin after 3 months of treatment were identified as potential risk factors for relapse among patients with pMN who achieved remission. Larger-scale studies are warranted for further validation.

## Introduction

Primary membranous nephropathy (pMN) is a common cause of adult idiopathic nephrotic syndrome (1). PMN is an antibody-mediated disorder characterized by subepithelial immune complex deposition along the glomerular basement membrane. In 70-80% of pMN cases, this results from autoantibodies targeting podocyte antigens, primarily the M-type phospholipase A2 receptor (PLA2R), with less frequent involvement of thrombospondin type-1 domain-containing 7A (THSD7A) or neural epidermal growth factor-like 1 (NELL1) (2, 3). While approximately one-third of patients achieve spontaneous remission with conservative management, another third progress to end-stage renal disease (ESRD) within 10 years (4, 5). Spontaneous remission may occur after years of persistent proteinuria (6).

Personalized treatment for pMN requires accurate prediction of disease course early in its progression, prior to initiating or adjusting immunosuppressive treatment. Traditional biomarkers include the magnitude and duration of proteinuria, and deterioration of renal function, reflected by increased serum creatinine or reduced creatinine clearance (5). Recent evidence supports that the measurement of anti-PLA2R antibodies titers may provide additional prognostic value. Anti-PLA2R antibody levels rise during clinically active disease and decline prior to clinical remission, supporting a serology-based approach for the treatment to PLA2R-related pMN (7, 8). High anti-PLA2R antibody levels at presentation are associated with poor clinical outcome (9). The MENTOR trial demonstrated that a 3-month composite evaluation of anti-PLA2R antibody and serum albumin levels best predicted therapeutic outcomes for cyclosporine or rituximab, outperforming baseline or single-marker assessments, with 6-month data offering no further predictive advantage (10).

Disease relapse is common in pMN, occurring in approximately 20%-30% of patients, with a reported median time to relapse of 6–20 months following remission (11–13). Sustained remission significantly improves renal survival, with even 3-month remission durations conferring prognostic benefit versus relapse (14). Previous studies have identified clinicopathological prognostic factors, including tubulointerstitial damage (15), glomerular lesions with focal segmental sclerosis (16), and advanced histological stage of electron-dense deposits (17). Immunosuppressive treatment induces a decline in anti-PLA2R antibodies before improvement in proteinuria, with immunologic remission typically preceding clinical remission by 6–12 months. Notably, incomplete antibody depletion at 6 months predicts relapse in rituximab-treated PLA2R-related MN (9). Current limitations include exclusive 6-month antibody measurements and insufficient integration of routine clinical parameters, such as proteinuria, albumin, and renal function (10). These evidence gaps contribute to the Kidney Disease: Improving Global Outcomes (KDIGO) recommendations being largely based on expert opinion rather than robust longitudinal serologic data.

However, anti-PLA2R antibody dynamics following remission and during relapsing disease remain poorly characterized. In this study, we retrospectively analyzed serial anti-PLA2R antibody levels in 154 Chinese patients with pMN who were followed up for at least 1 year after treatment initiation. We furthermore evaluated the longitudinal measurement of clinical variables and anti-PLA2R antibody levels at baseline, 3 months, and 6 months and their association with relapse after remission.

## Subjects and methods

### Study population

This retrospective study was conducted at Jining No.1 People’s Hospital between January 2019 and October 2024. Patients with biopsy-proven pMN were followed up via telephone and the electronic medical record system. The following inclusion criteria were used: (1) biopsy-proven pMN; (2) baseline anti-PLA2R antibody levels ≥ 14 RU/ml; (3) 24-hour urinary protein ≥ 3.5 g/d at the time of biopsy and receiving immunosuppressive therapy; (4) complete clinical and pathological data; (5) follow-up time ≥ 12 months; and (6) age ≥ 18 years old. Patients with secondary MN, liver impairment, severe infections or malignancy were excluded. This study was approved by the ethics committee of Jining No.1 People’s Hospital (No. 2025-IIT-029).

### Data collection

The demographic variables included sex, age, and clinical variables included follow-up duration, treatment regimen, and comorbidities such as hypertension, diabetes mellitus, and coronary artery disease. Laboratory data were collected at baseline (the date of the onset of treatment), and at 3, 6, 9, and 12 months of follow-up. These included 24-hour urinary protein, anti-PLA2R antibody titers, serum creatinine, estimated glomerular filtration rate (eGFR), and serum levels of albumin, globulin, cholesterol, and uric acid. Based on Ehrenreich-Churg classification, pMN was divided into four stages: stage I, stage II, stage III and stage IV (18). The renal pathology included interstitial fibrosis/tubular atrophy, global glomerulosclerosis and segmental glomerulosclerosis. Serum anti-PLA2R antibodies were measured with a commercial quantum dot fluorescence immunoassay kit by indirect immunofluorescence assay (Vazyme Biotech, Nanjing, China). The cutoff for anti-PLA2R antibody levels was set at ≥ 14 RU/ml, as this represents the manufacturer’s threshold for seropositivity and is consistent with the 2021 KDIGO guidelines (19).

Patients were assigned to three groups based on their treatment regimen: RTX (rituximab-based), CTX + GC (cyclophosphamide plus glucocorticoids), and CNI ± GC (calcineurin inhibitors with or without glucocorticoids). In the CNI group, oral tacrolimus (0.05 mg/kg/d, trough 4-8 ng/ml) or cyclosporine (3-5 mg/kg/d, trough 125-175 ng/ml) is tapered after 6–9 months to minimum effective dose for ≥ 12 months. For the CTX group, IV cyclophosphamide (500–750 mg/m² monthly for 6 months, then every 2–3 months) was combined with prednisone. RTX treatment (1,000 mg × 2 doses, 2 weeks apart) was followed by individualized retreatment based on B-cell recovery, anti-PLA2R, and remission status (20).

### Outcomes

Treatment responses included both complete remission (CR) and partial remission (PR). CR was defined by a proteinuria value of ≤ 0.3 g/d, along with a serum albumin level of at least 35 g/L, a stable eGFR (21), and anti-PLA2R antibody titers < 14RU/ml. PR was defined by a proteinuria value of > 0.3 but < 3.5 g/d plus a 50% reduction from its baseline and anti-PLA2R antibody titers < 14RU/ml, along with improvement in the serum albumin concentration, a stable eGFR (21). Treatment responses (either CR or PR) were assessed at 12 months after treatment initiation. Patients who achieved CR or PR within 12 months were classified as the remission cohort. The 12-month time point was used as the prespecified eligibility window and was not considered the date of remission for all patients. The definition of relapse was a proteinuria value ≥ 3.5 g/d after documented complete or partial remission (22). Anti-PLA2R antibody seronegativity was defined as an antibody level below the assay-specific threshold of 14 RU/mL for negativity. Anti-PLA2R antibody seropositivity was defined as an antibody level ≥ 14 RU/mL.

### Statistical analysis

Data were analyzed by SPSS (version 26.0), GraphPad software (version 9.0), and R software (version 4.5.1). Normality of continuous variables was assessed using the Kolmogorov-Smirnov test. Variables with a normal distribution were presented as the mean ± standard deviation (SD) and were compared by using Student’s t test. Non-normally distributed data were expressed as median with interquartile range (IQR) and compared by using Mann-Whitney U test. Categorical variables were presented as frequencies and percentages and compared by using the chi-square test or Fisher’s exact test, as appropriate.

Univariable and multivariable Cox proportional-hazards regression analyses were subsequently performed to assess the associations between these variables and the risk of relapse. Model fit was compared across different landmark time points and variable combinations using the Akaike information criterion (AIC) and Harrell’s C-index. Receiver-operating characteristic (ROC) curves and the area under the curve (AUC) were used to evaluate the predictive performance of individual parameters for relapse. Kaplan–Meier curves were analyzed to evaluate relapse-free survival, and the log-rank test was used. Time zero was defined as the date of confirmed remission, and patients without relapse were censored at the date of last follow-up. For each patient, the date of first documented remission was recorded individually, and relapse was defined as disease recurrence occurring after that date. Sensitivity analyses were conducted by excluding patients receiving specific immunosuppressive therapies. *P*-values <0.05 were defined as statistically significant.

## Results

### Clinical characteristics

A total of 154 patients with PLA2R-associated pMN were enrolled in the study (**Supplementary Figure 1**). The median follow-up time was 26 months (IQR 18.00-34.00). 96 patients (62.34%) achieved remission (either PR or CR) of nephrotic syndrome at 12 months with immunosuppressive treatment and were classified as the remission group for subsequent relapse analysis. The median time from treatment initiation to first documented remission was 6 months (IQR 5-9) in the remission group. The remaining 58 patients were defined as the non-remission group. The remission group showed significantly lower 24-hour urinary protein and serum creatinine, and significantly higher eGFR, serum albumin, and globulin levels than the non-remission group (all *p* < 0.05, **Table 1**). At baseline, the remission group exhibited significantly lower anti-PLA2R titers [45.50 (23.00-76.75) vs. 125.00 (63.00-223.25) RU/mL, *p* < 0.001] and a lower prevalence of titers ≥ 20 RU/mL (78.13% vs. 93.10%, *p* = 0.01) compared to the non-remission group. A significantly lower proportion of males was also observed in the remission group, compared to the non-remission group (48.96% vs. 68.96%, *p* = 0.02). No significant differences were observed in other characteristics.

**Table 1:** The characteristics of pMN patients with remission or non-remission at baseline.

Characteristics	Overall cohort (n=154)	Remission (n=96)	Non-remission (n=58)	*P* value
Age, years	54.00 (43.00-60.00)	54.00 (45.00-59.00)	55.00 (42.75-61.25)	0.30
Male sex, n (%)	87 (56.49)	47 (48.96)	40 (68.96)	0.02
Hypertension, n (%)	82 (53.25)	51 (53.12)	31 (53.45)	1.0
Diabetes, n (%)	24 (15.58)	15 (15.62)	9 (15.52)	1.0
Coronary artery disease, n (%)	12 (7.79)	6 (6.25)	6 (10.34)	0.37
Laboratory parameters				
Proteinuria, g/d	8.02 (6.21-9.49)	7.68 (5.81-9.06)	8.46 (6.64-11.02)	0.02
Serum globulin, g/L	22.93 ± 3.58	23.38 ± 3.38	22.17 ± 3.79	0.03
Serum albumin, g/L	24.45 (21.95-27.50)	25.20 (22.42-28.40)	23.10 (20.55-25.50)	<0.001
Serum cholesterol, mmol/L	7.65 (6.30-9.44)	7.58 (6.08-9.39)	7.95 (6.68-9.69)	0.37
Serum uric acid, μmol/L	334.00 (285.00-409.50)	334.00 (274.75-401.25)	336.00 (285.00-439.75)	0.47
Serum creatinine, μmol/L	66.80 (53.65-81.00)	63.75 (52.32-74.75)	73.85 (62.30-83.50)	0.002
eGFR, ml/min per 1.73 m2	101.10 (91.66-109.65)	102.63 (92.50-113.18)	98.65 (85.52-104.80)	0.01
CD19,/μL	287.14 (165.75-414.11)	237.35 (151.37-364.94)	312.18 (204.18-579.74)	0.15
PLA2R antibody level, RU/mL	58.50 (30.00-129.75)	45.50 (23.00-76.75)	125.00 (63.00-223.25)	<0.001
PLA2R antibody levels, n (%)				
14–20 RU/mL	25 (16.23)	21 (21.87)	4 (6.90)	0.01
≥ 20 RU/mL	129 (83.77)	75 (78.13)	54 (93.10)	
Treatment with ACEI/ARB, n (%)	98 (63.64)	59 (61.46)	39 (67.24)	0.49
Glucocorticoid, n (%)	108 (70.13)	66 (68.75)	42 (72.41)	0.72
Treatment allocation, n (%)				
RTX	43 (27.92)	28 (29.17)	15 (25.86)	0.88
CTX+GC	48 (31.17)	30 (31.25)	18 (31.03)	
CNIs ± GC	63 (40.91)	38 (39.58)	25 (43.11)	
Prior immunosuppressive drugs^*^, n (%)	14 (9.09)	10 (10.42)	4 (6.90)	0.57

*Prior immunosuppressive drugs including cyclophosphamide and calcineurin inhibitors. P value < 0.05 were considered as significant. pMN, primary membranous nephropathy; eGFR, estimated glomerular filtration rate; ACEI/ARB, angiotensin converting enzyme inhibitors/angiotensin receptor blocker; RTX, rituximab; CTX, cyclophosphamide; GC, glucocorticoids; CNIs, calcineurin inhibitors; PR, partial remission; CR, complete remission.

### Comparisons between pMN patients with relapse and non-relapse

The clinical characteristics of the remission cohort were summarized in **Table 2**. The median follow-up time was 32 months (IQR 25.25-36.00). The median age at diagnosis was 54 years (IQR 45-59). In total, 28 patients (29.17%) received RTX, 30 (31.25%) received CTX + GC, and 38 (39.58%) received CNIs ± GC. And 59 (61.46%) patients received ACEI/ARB therapy. Ten (10.42%) patients had a history of prior immunosuppressive treatment including CTX or CNI. Comorbid conditions included hypertension in 51 patients (53.12%), diabetes mellitus in 15 (15.62%), and coronary artery disease in 6 (6.25%). Median 24-hour proteinuria was 7.68 g/day (IQR 5.81-9.06), serum albumin was 25.20 g/L (IQR 22.42-28.40) and serum cholesterol was 7.58 mmol/L (IQR 6.08-9.39). Baseline renal function was within the normal range across the cohort, with a median eGFR of 102.63 ml/min/1.73 m^2^ (IQR 92.50-113.18). Global glomerulosclerosis was observed in 44 patients (45.83%), segmental glomerulosclerosis in 21 (21.87%), and mild interstitial fibrosis/tubular atrophy was present in 93 (96.88%) patients (**Supplementary Table 1**). Based on the Ehrenreich–Churg classification, 22 patients (22.92%) were categorized as Stage I, 60 (62.50%) as Stage II, and 14 (14.58%) as Stage III.

**Table 2:** Baseline characteristics of pMN patients achieving remission, stratified by subsequent relapse status.

Characteristics	Overall cohort (n=96)	Relapse (n=17)	Non-relapse (n=79)	*P* value
Age, years	54.00 (45.00-59.00)	47.00 (34.50-53.50)	54.00 (47.00-60.00)	0.04
Sex (Male), n (%)	47 (48.96)	9 (52.94)	38 (48.10)	0.79
Hypertension, n (%)	51 (53.12)	8 (47.06)	43 (54.43)	0.60
Diabetes, n (%)	15 (15.62)	1 (5.88)	14 (17.72)	0.29
Coronary artery disease, n (%)	6 (6.25)	1 (5.88)	5 (6.33)	1.0
Laboratory parameters				
Proteinuria, g/d	7.68 (5.81-9.06)	7.02 (5.85-8.33)	7.78 (5.70-9.27)	0.38
Serum globulin, g/L	23.38 ± 3.38	23.28 ± 2.51	23.40 ± 3.56	0.74
Serum albumin, g/L	25.20 (22.42-28.40)	24.90 (22.25-27.90)	25.30 (22.60-28.70)	0.50
Serum cholesterol, mmol/L	7.58 (6.08-9.39)	7.85 (7.04-11.20)	7.47 (5.93-9.25)	0.18
Serum uric acid, μmol/L	334.00 (274.75-401.25)	399.00 (260.50-465.50)	330.00 (280.00-389.50)	0.27
Serum creatinine, μmol/L	63.75 (52.32-74.75)	60.20 (50.25-77.70)	64.70 (53.00-74.00)	0.62
eGFR, ml/min per 1.73 m2	102.63 (92.50-113.18)	111.50 (101.06-119.99)	101.40 (91.80-112.55)	0.05
CD19,/μL	237.35 (151.37-364.94)	370.43 (216.93-523.92)	237.35 (145.54-343.95)	0.58
PLA2R antibody level, RU/mL	45.50 (23.00-77.75)	71.00 (57.10-106.57)	37.00 (18.00-65.00)	<0.001
PLA2R antibody levels, n (%)				
14–20 RU/mL	21 (21.87)	0	21 (26.58)	0.02
≥ 20 RU/mL	75 (78.13)	17 (100)	58 (73.42)	
Treatment responses, n (%)				
PR	63 (65.63)	14 (82.35)	49 (62.03)	0.16
CR	33 (34.37)	3 (17.65)	30 (37.97)	
Treatment with ACEI/ARB, n (%)	59 (61.46)	7 (41.18)	52 (65.82)	0.10
Glucocorticoid, n (%)	66 (68.75)	15 (88.23)	51 (64.56)	0.08
Treatment allocation, n (%)				
RTX	28 (29.17)	2 (11.77)	26 (32.91)	0.13
CTX+GC	30 (31.25)	5 (29.41)	25 (31.65)	
CNIs ± GC	38 (39.58)	10 (58.82)	28 (35.44)	
Prior immunosuppressive drugs, n (%)	10 (10.42)	1 (5.88)	9 (11.39)	0.68

By the end of follow-up, 17 (17.71%) patients experienced disease relapse in the remission group, and the median times to relapse post-treatment and post-remission were 23 months (IQR, 14.5-29.0) and 15.5 months (IQR, 6.0-22.5), respectively. Patients in the relapse group were significantly younger than those in the non-relapse group [47 (34.5-53.5) vs. 54 (47-60) years; *p* = 0.04]. The relapse group showed significantly higher baseline anti-PLA2R antibody levels [71.00 (IQR 57.10-106.57) vs. 37.00 (IQR 18.00-65.00) RU/mL; *p* < 0.001] than the non-relapse group. The proportion of patients with anti-PLA2R antibody titers ≥ 20 RU/mL was significantly higher in the relapse group compared to the non-relapse group (100% vs. 73.42%, *p* = 0.02). There were no significant differences in initial immunosuppressive therapy between groups. In the relapse group, 10 (58.82%) patients received CNI, 5 (29.41%) received CTX, and 2 (11.77%) received RTX. No other significant differences were observed between the relapse and non-relapse groups in terms of the demographic, clinical, or pathological characteristics (**Table 2**; **Supplementary Table 1**).

### Anti-PLA2R antibody dynamics between relapse and non-relapse groups

We further investigated the changes of anti-PLA2R antibody and renal function-related parameters over time. Anti-PLA2R antibody and serum albumin levels showed considerable overlap between patients with and without relapse at baseline, became clearly differentiated by relapse status at 3 and 6 months, and converged again at 9 months (**Figures 1A, B**). Similarly, the proportion of patients achieving anti-PLA2R antibody seronegativity differed markedly by subsequent relapse status at 3 and 6 months (**Figure 1C**). At 3 months, the relapse group had significantly higher anti-PLA2R antibody levels than the non-relapse group [20.00 (14.50-25.00) vs 10.00 (5.00-15.00) RU/mL, *p* < 0.001; **Table 3**]. Notably, only 17.65% of relapsed patients achieved anti-PLA2R levels < 14 RU/mL, compared with 74.68% of the non-relapse group, whereas a higher proportion of relapsed patients reached levels ≥ 20 RU/mL (52.94% vs. 15.19%, *p* < 0.001; **Table 3**). In contrast, relapse patients exhibited a significantly greater absolute reduction in anti-PLA2R antibody levels over the first 3 months (**Table 3**), which is explained by their higher baseline anti-PLA2R titers. Moreover, serum albumin levels at 3 months were significantly lower in the relapse group than in the non-relapse group [30.80 (29.40-33.00) vs. 34.20 (31.90-37.20) g/L, *p* < 0.001] and the increase in albumin levels over 3 months was also significantly smaller in patients with relapse (**Table 3**). In contrast, neither 3-month proteinuria levels nor the reduction from baseline differed significantly between the relapse and non-relapse groups (both p > 0.05, **Table 3**).

**Figure 1:**
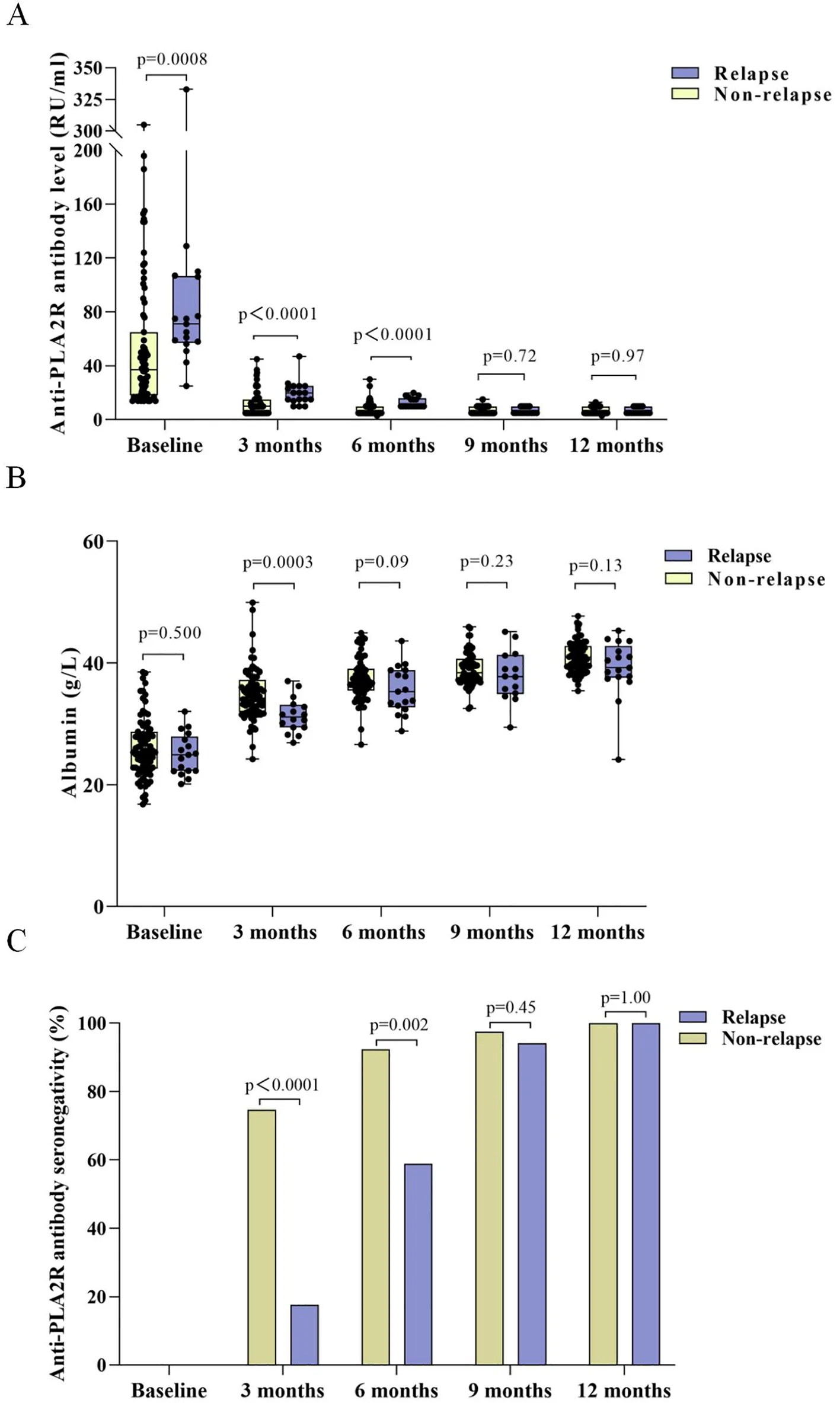
Anti-PLA2R antibody and albumin levels in patients with primary membranous nephropathy (pMN) over time based on relapse status. **(A)** Anti-PLA2R antibody levels over time. **(B)** Serum albumin levels over time. Data in the figure are expressed as median with interquartile range (IQR). **(C)** Percentage of patients achieving anti-PLA2R antibody seronegativity (< 14 RU/mL) over time.

**Table 3:** Clinical characteristics of the cohort at 3 months post treatment.

Characteristics at 3 months	Overall cohort (n=96)	Relapse (n=17)	Non-relapse (n=79)	*P* value
Proteinuria at 3 mo, g/d	4.52 (3.58-5.53)	4.53 (3.16-5.53)	4.30 (3.60-5.54)	0.77
Proteinuria change from baseline to 3 mo (decrease), g/d	3.22 (2.02-4.44)	2.75 (1.82-3.49)	3.40 (2.22-4.62)	0.12
Albumin at 3 mo, g/L	33.75 (31.50-36.17)	30.80 (29.40-33.00)	34.20 (31.90-37.20)	<0.001
Albumin change from baseline to 3 mo (increase), g/L	7.75 (5.90-10.85)	6.40 (4.80-7.95)	8.30 (6.40-11.70)	0.02
eGFR at 3 mo, ml/min per 1.73 m^2^	100.39 (90.52-110.75)	110.30 (101.11-117.45)	99.40 (90.23-109.10)	0.01
eGFR change from baseline to 3 mo (increase), ml/min per 1.73 m^2^	1.95 (-2.77-7.19)	-0.92 (-5.08-8.17)	2.00 (-2.00-7.20)	0.44
CD19 at 3 mo,/μL	2.48 (1.70-4.95)	5.05 (3.40-6.70)	2.35 (1.70-4.85)	0.26
CD19 change from baseline to 3 mo (decrease),/μL	234.10 (149.65-362.00)	365.35 (213.50-517.20)	234.10 (143.15-338.80)	0.58
PLA2R antibody level at 3 mo, RU/mL	10.00 (5.00-15.00)	20.00 (14.50-25.00)	10.00 (5.00-15.00)	<0.001
PLA2R antibody levels at 3 mo, n (%)				
< 14 RU/mL	62 (64.58)	3 (17.65)	59 (74.68)	<0.001
14–20 RU/mL	13 (13.54)	5 (29.41)	8 (10.13)	
≥20 RU/mL	21 (21.88)	9 (52.94)	12 (15.19)	
PLA2R antibody change from baseline to 3 mo (decrease), RU/mL	32.50 (15.00-60.75)	51.00 (35.50-86.00)	26.00 (13.00-54.00)	0.01

Mo, month.

At 6 months, anti-PLA2R antibody levels remained significantly elevated in the relapsing group compared with the non-relapsing group [10.00 (10.00-16.00) vs. 5.00 (5.00-10.00) RU/mL, *p* < 0.001; **Supplementary Table 2**]. The proportion of patients achieving anti-PLA2R antibody < 14 RU/mL at 6 months was significantly lower in the relapse group than in the non-relapse group (58.82% vs. 92.41%, p < 0.001). Similarly, the absolute change in anti-PLA2R antibody levels from baseline differed significantly between the two groups [60.00 (44.50-89.50) vs. 31.00 (13.00-54.00) RU/mL, *p* = 0.002].

### Predictors for relapse

Subsequently, we integrated these parameters at each time point with clinical variables to evaluate their associations with relapse risk using univariable and multivariable Cox regression analyses. In the baseline model, higher anti-PLA2R antibody levels showed a trend toward an increased relapse risk (HR 1.01, 95% CI: 1.00-1.01, *p* = 0.08; **Supplementary Table 3**). In the 3-month landmark model, anti-PLA2R antibody seropositivity was significantly associated with a higher relapse risk (HR 9.44, 95% CI 2.69-33.21; p < 0.001), whereas a greater increase in serum albumin was associated with a lower relapse risk (HR 0.85, 95% CI 0.74-0.98; p = 0.03; **Table 4**). In the 6-month landmark analysis, anti-PLA2R antibody seropositivity remained significantly associated with relapse (HR 4.76, 95% CI 1.80-12.61; p = 0.002), whereas the albumin change from baseline to 6 months was not significantly associated with relapse risk (HR 0.97, 95% CI 0.90-1.04; p = 0.39; **Supplementary Table 4**). Treatment regimens, including calcineurin inhibitors, rituximab, and cyclophosphamide, were not significantly associated with relapse risk in the univariable models (all p > 0.05; **Table 4**).

**Table 4:** Univariable and multivariable Cox regression models for relapse using risk factors at baseline and at 3 months.

Risk factors	Univariable model		Multivariable model	
	HR (95% CI)	*P* value	HR (95% CI)	*P* value
PLA2R antibody change from baseline to 3 mo (per RU/ml, decrease)	1.01(1.00-1.01)	0.14	–	–
Anti-PLA2R antibody seropositivity at 3 mo	10.35(2.97-36.07)	<0.001	9.44(2.69-33.21)	<0.001
PLA2R antibody level at baseline (per RU/ml)	1.01(1.00-1.01)	0.08	–	–
Proteinuria change from baseline to 3 mo (per g/d, decrease)	0.80(0.60-1.06)	0.11	–	–
Proteinuria at baseline (per g/d)	0.89(0.72-1.10)	0.28	–	–
Albumin change from baseline to 3 mo (per g/L, increase)	0.83(0.71-0.95)	0.009	0.85(0.74-0.98)	0.03
Albumin at baseline (per g/L)	0.97(0.87-1.08)	0.58	–	–
Treatment allocation (versus cyclophosphamide)				
RTX	0.46(0.09-2.38)	0.36	–	–
CNIs	1.62(0.55-4.76)	0.38	–	–

Anti-PLA2R antibody seropositivity at 3 months was defined as anti-PLA2R antibody levels ≥ 14 RU/mL, using the manufacturer-specified seropositivity threshold. The reference group was patients with levels < 14 RU/mL (seronegative). HR, hazard ratio; CI, confidence interval.

In treatment-specific sensitivity analyses, anti-PLA2R antibody seropositivity at 3 months remained significantly associated with relapse and the albumin increments from baseline to 3 months remained associated with a lower relapse risk (**Supplementary Table 5**).

### Multivariable predictive models for relapse

At each landmark time, measures of model fit (AIC) and discrimination (Harrell’s C-index) were used to compare the multivariable models. The 3-month multivariable model that included anti-PLA2R antibody seropositivity and albumin increment achieved the lowest AIC of 123.41 and the highest C-index of 0.83, compared with the 3-month univariable model that included only anti-PLA2R antibody seropositivity (AIC 126.72, C-index 0.78) and the 6-month multivariable model (AIC 138.84, C-index 0.66; **Supplementary Table 6**). At the 3-month landmark, the multivariable Cox model incorporating 3-month anti-PLA2R antibody seropositivity and albumin increment demonstrated an AUC of 0.83, compared with 0.78 for the univariable model that included only anti-PLA2R antibody seropositivity (**Figure 2A**). In contrast, at the 6-month landmark, the multivariable model showed no improvement over the univariable model, with an AUC of 0.66 vs. 0.67 (**Figure 2B**).

**Figure 2:**
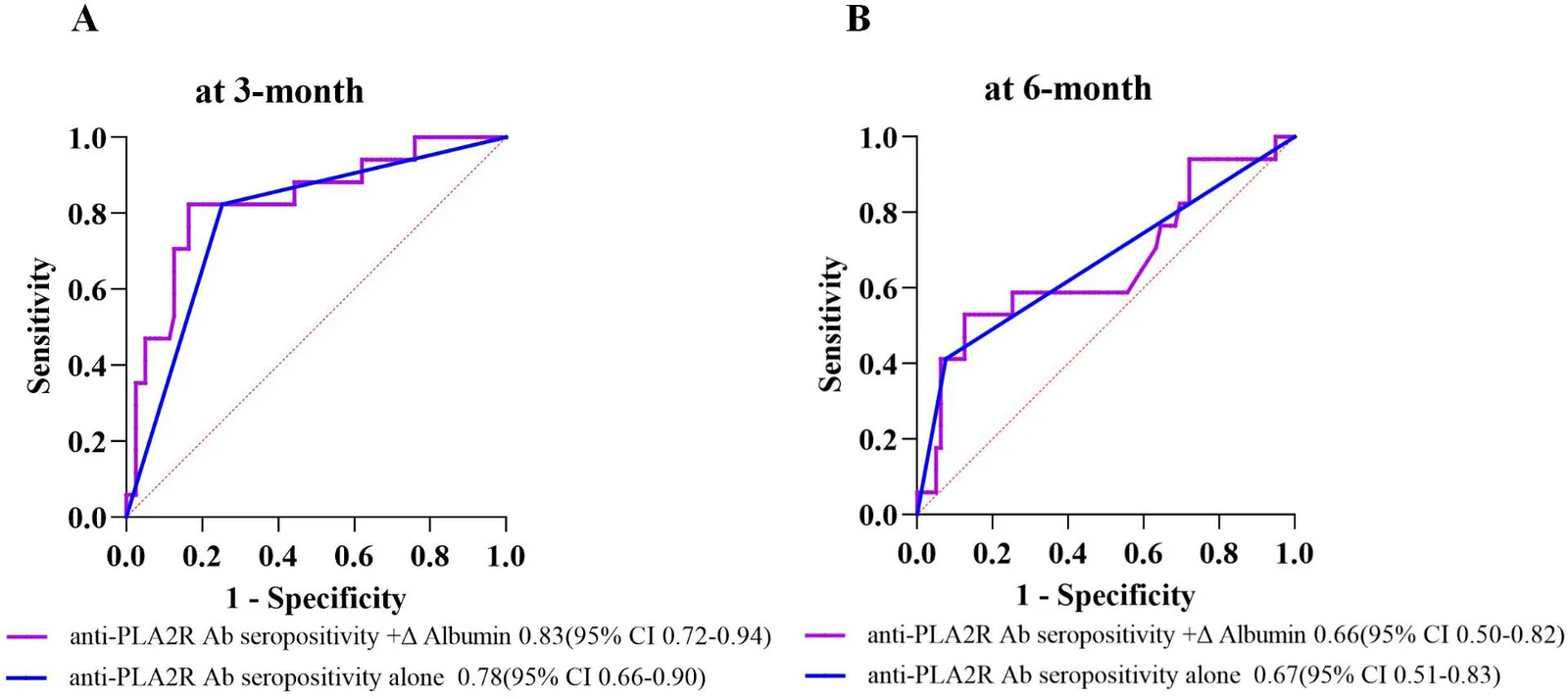
Receiver operating characteristic (ROC) curves for pMN relapse prediction at different landmark time points. **(A, B)** Comparison of ROC curves between the univariable model (anti-PLA2R antibody seropositivity alone) and the multivariable model (anti-PLA2R antibody seropositivity + Δ Albumin) at 3 months **(A)** and 6 months **(B)**. CI, confidence interval; Δ Albumin, change in serum albumin from baseline to the corresponding landmark time.

We further evaluated the clinical utility of 3-month anti-PLA2R antibody seropositivity by performing Kaplan–Meier analysis stratified by anti-PLA2R antibody levels (<14 vs. ≥14 RU/mL) at 3 months. Patients with anti-PLA2R antibody seropositivity at 3 months had significantly lower relapse-free survival than those with seronegative status (p < 0.001; **Supplementary Figure 2**).

## Discussion

PMN is a common form of primary glomerulonephritis in our country, and its proportion among primary glomerular diseases has been increasing in recent years (23). PMN is a slowly progressive disorder; approximately 20–30% of patients develop ESRD over several years. Achieving remission is strongly associated with improved renal survival both at initial presentation and after relapse (24). Although most patients can achieve clinical remission through supportive care or immunosuppressive therapy, disease relapse remains a significant clinical challenge. Relapsing patients are at higher risk of developing focal segmental glomerulosclerosis, persistent proteinuria, and progression to ESRD (25). Previous studies have often focused on baseline clinical characteristics, such as renal function, anti-PLA2R antibody levels, and renal pathology, to predict remission and relapse risk in pMN (12, 26, 27). However, changes in anti-PLA2R antibody levels and renal function within the first 3 to 6 months of treatment may also have prognostic value. Early identification of relapse is crucial for preventing loss of kidney function and reducing the duration of high-risk periods.

In our cohort of pMN patients treated with cyclophosphamide, rituximab, or CNI, approximately 60% achieved complete or partial remission within 12 months, consistent with previous reports (12, 20). Remission was associated with higher baseline eGFR, higher serum albumin and globulin, lower anti-PLA2R antibody titers, as well as lower proteinuria and serum creatinine, aligning with earlier findings (5). Although immunosuppressive agents may influence the prognostic value of anti-PLA2R antibodies, no significant differences in remission rates were observed among treatment groups in this study. Additionally, rituximab was more effective than CNI and comparable to CTX in inducing remission, consistent with a recent single-center retrospective study (20). These results are also supported by a multicenter retrospective study that found no significant difference in remission rates between RTX and intermittent intravenous CTX with corticosteroids over two years (28).

The cumulative relapse rate in our study was 17.71% by the end of follow-up, with a median time to relapse after remission of 15.5 months. This finding is consistent with reports from a Chinese single-center study and a Japanese multicenter study, which showed 2-year cumulative relapse rates of 26.5% and 22%, respectively (12, 29). Relapse occurred in 10 of 38 (26%) patients in the CNI group, a numerically higher proportion than in the CTX and RTX groups, although the difference among treatment groups was not statistically significant, supporting previous observations that CNI may be associated with higher risks of proteinuria relapse and eGFR decline (20). In our cohort, relapse was associated with higher baseline anti-PLA2R antibody levels, although baseline antibody levels did not reach independent significance in the landmark Cox models, consistent with the established role of anti-PLA2R antibodies as prognostic biomarkers in pMN (30). Incomplete depletion of anti-PLA2R antibodies by month 6 has been linked to relapse in patients treated with RTX (9), and pre-transplant antibody levels predict recurrence in allograft membranous nephropathy (31). Although previous studies identified hyperuricemia, CRP, remission status, and treatment regimen as predictors of relapse (12), the distinguishing baseline feature in this cohort were younger age and higher anti-PLA2R antibody levels in pMN patients with relapse.

In our study, anti-PLA2R antibody seropositivity at 3 months was independently associated with relapse risk. Given the inherent dependence of absolute antibody changes on baseline antibody burden, we focused on seroconversion as a more meaningful measure and clinical utility of the early immunologic response. Although patients who subsequently relapsed appeared to exhibit a greater absolute reduction in anti-PLA2R antibody levels at 3 months, this finding was likely attributable to their higher titers. In contrast, anti-PLA2R antibody seroconversion at 3 months provided a clinically interpretable and statistically parsimonious indicator of early immunologic response and remission status.

Importantly, combining anti-PLA2R antibody status with serum albumin further improved relapse prediction, suggesting that integrating early immunologic and biochemical responses may provide a more comprehensive assessment of treatment effectiveness. Specifically, the 3-month landmark model incorporating anti-PLA2R antibody seropositivity and the increase in serum albumin from baseline demonstrated improved predictive performance across multiple complementary analyses. In contrast, our exploratory analysis showed that extending the assessment to 6 months conferred no additional prognostic benefit over the 3-month model. These findings support 3 months as a potentially informative time point for early risk stratification and may facilitate timely adjustment of treatment in patients with persistent antibody positivity and limited albumin recovery. This supports the clinical relevance of early monitoring and is consistent with evidence from the MENTOR trial (10), which highlighted the value of 3-month anti-PLA2R antibody and albumin assessment for evaluating therapeutic response in pMN.

Our study offers insight into the immunologic trajectory of PLA2R-mediated MN post-treatment. Multiple studies indicate that antibody levels decline earlier than proteinuria after immunosuppression, and immunologic remission precedes clinical remission (7, 32). At 6 months, anti-PLA2R antibody seroconversion remained predictive of relapse. The 3-month anti-PLA2R antibody seroconversion status, combined with albumin increment, effectively stratified patients into distinct risk groups and showed potential for predicting relapse. Both this study and the MENTOR trial suggest that 3-month assessment of albumin and antibody levels may be useful for early evaluation of treatment response and prediction of long-term outcomes (10). While the MENTOR trial evaluated changes in anti-PLA2R antibody levels as a continuous measure, our study extends this by demonstrating that anti-PLA2R antibody seroconversion can serve as a simple, practical alternative for risk stratification.

The prognostic importance of anti-PLA2R antibody seroconversion is further supported by previous studies. In the GEMRITUX trial, an early decline in anti-PLA2R antibody levels within the first 3 months of rituximab therapy was the strongest predictor of remission at 6 months (33). In a prospective cohort of patients with aPLA2R-associated pMN, Ramachandran et al. showed that serological remission consistently preceded clinical remission (34). Consistent with these observations, early anti-PLA2R antibody seroconversion was associated with sustained remission and a lower risk of relapse in our cohort, extending the prognostic relevance of early antibody monitoring beyond remission induction to the prediction of post-remission relapse.

Our study has several limitations. First, its single-center retrospective design and relatively small sample size necessitate external validation through future prospective, long-term, multicenter cohort studies. Second, the limited number of relapse events and single-center design still carry a potential risk of overfitting. Third, anti-PLA2R antibodies were measured using a quantum dot fluorescence immunoassay. While this assay demonstrated high concordance with traditional ELISA in our internal validation and prior research (35), its use may hinder direct comparability with ELISA-based studies; furthermore, antibody titers below 5 RU/mL could not be further quantified. Finally, several potential relapse-associated factors were not evaluated, including anti-rituximab antibodies (36), and early B-cell repopulation (37).

## Conclusion

In conclusion, among anti-PLA2R antibody-positive pMN patients receiving immunosuppressive therapy, anti-PLA2R antibody seropositivity at 3 months (anti-PLA2R ≥14 RU/mL) and blunted albumin increase during the first 3 months of treatment may be potential risk factors of proteinuria relapse after remission. Combining both markers showed promising predictive value in our exploratory study, and the 3-month assessment demonstrated favorable model performance compared with the 6-month assessment. Larger-scale studies with external validation are warranted to confirm these findings.

## Data Availability

The original contributions presented in the study are included in the article/**Supplementary Material**. Further inquiries can be directed to the corresponding authors.
