# Supplementary material for: Anti-PLA2R antibody levels and clinical risk factors for predicting relapse in primary membranous nephropathy
Source: Front Immunol. 2026 Sep 25;17:1780071. doi: 10.3389/fimmu.2026.1780071 (PMC13649681; doi:10.3389/fimmu.2026.1780071)
Supplement: Supplementary file 1 [file DataSheet1.pdf]

Supplemental Materials

Anti-PLA2R antibody levels and clinical risk factors for predicting relapse in primary membranous nephropathy

Hongju Wang[1], Huijuan Ma[1]*, Huijing Wang[2]*

[1] Department of Nephrology, Jining No.1 People's Hospital, Jining, China, [2]Rheumatology, Renji Hospital, Shanghai Jiao Tong University School of Medicine, Shanghai, China

***Correspondence:** Huijing Wang, Email: whj30813@163.com; and Huijuan Ma, Email: ythuijuan@163.com

Table of Contents

Supplementary Figures

Supplementary Figure 1: Patient flowchart.

Supplementary Figure 2: Kaplan-Meier curves for relapse-free survival from the date of remission, stratified by 3-month anti-PLA2R antibody status.

Supplementary Tables

Supplementary Table 1: Histological Characteristics in pMN patients with remission at baseline

Supplementary Table 2: Clinical characteristics of the cohort at 6 months post-treatment

Supplementary Table 3: Univariable and multivariable Cox regression models for relapse using risk factors at baseline

Supplementary Table 4: Univariable and multivariable Cox regression models for relapse using risk factors at baseline and at 6 months

Supplementary Table 5: Treatment-specific sensitivity analyses of multivariable Cox regression models for relapse

Supplementary Table 6: Model fit comparison at different landmark time points

Supplementary Figure 1

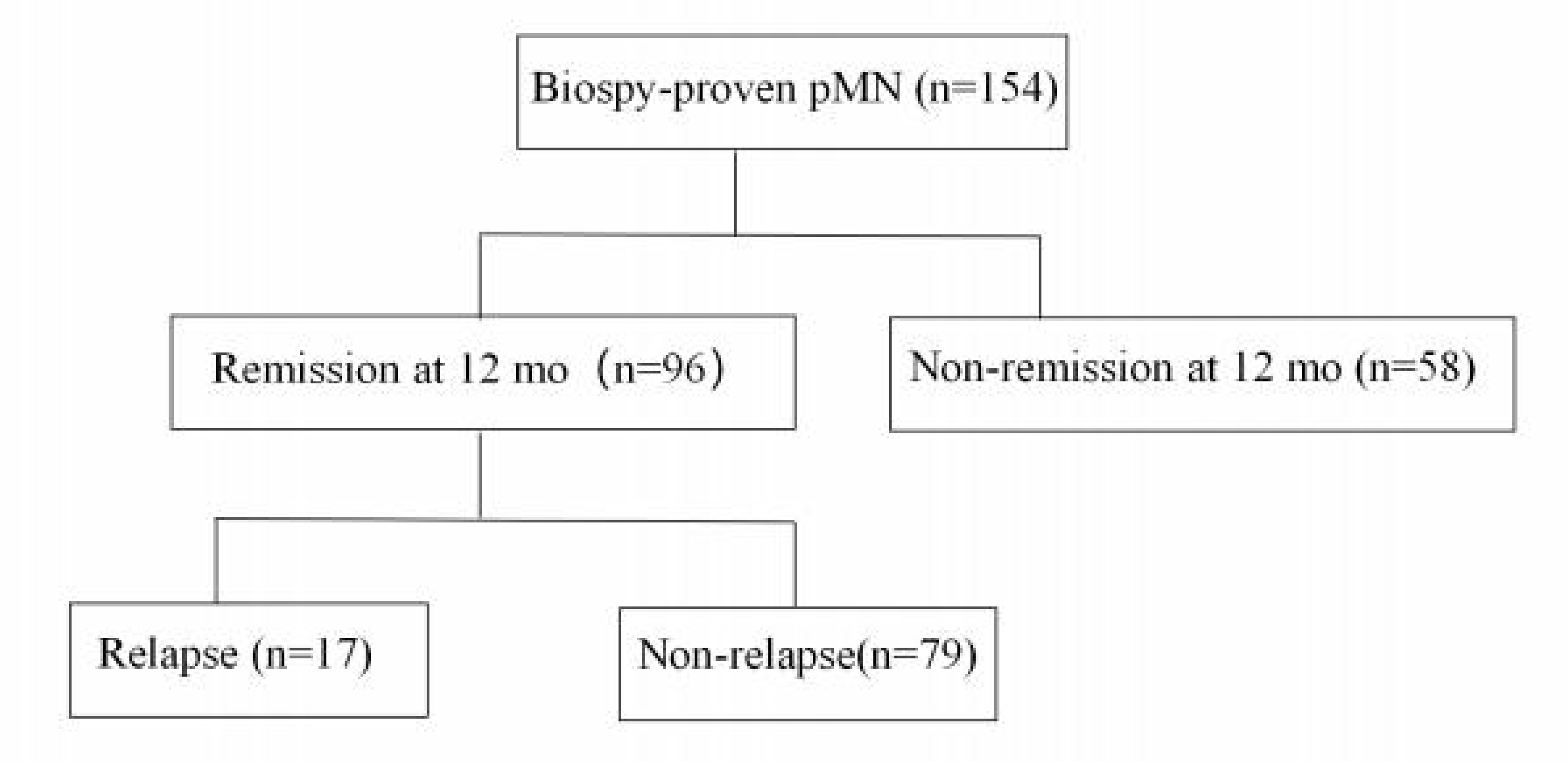

Supplementary Figure 1. Patient flowchart. pMN, primary membranous nephropathy; mo, months.

Supplementary Figure 2

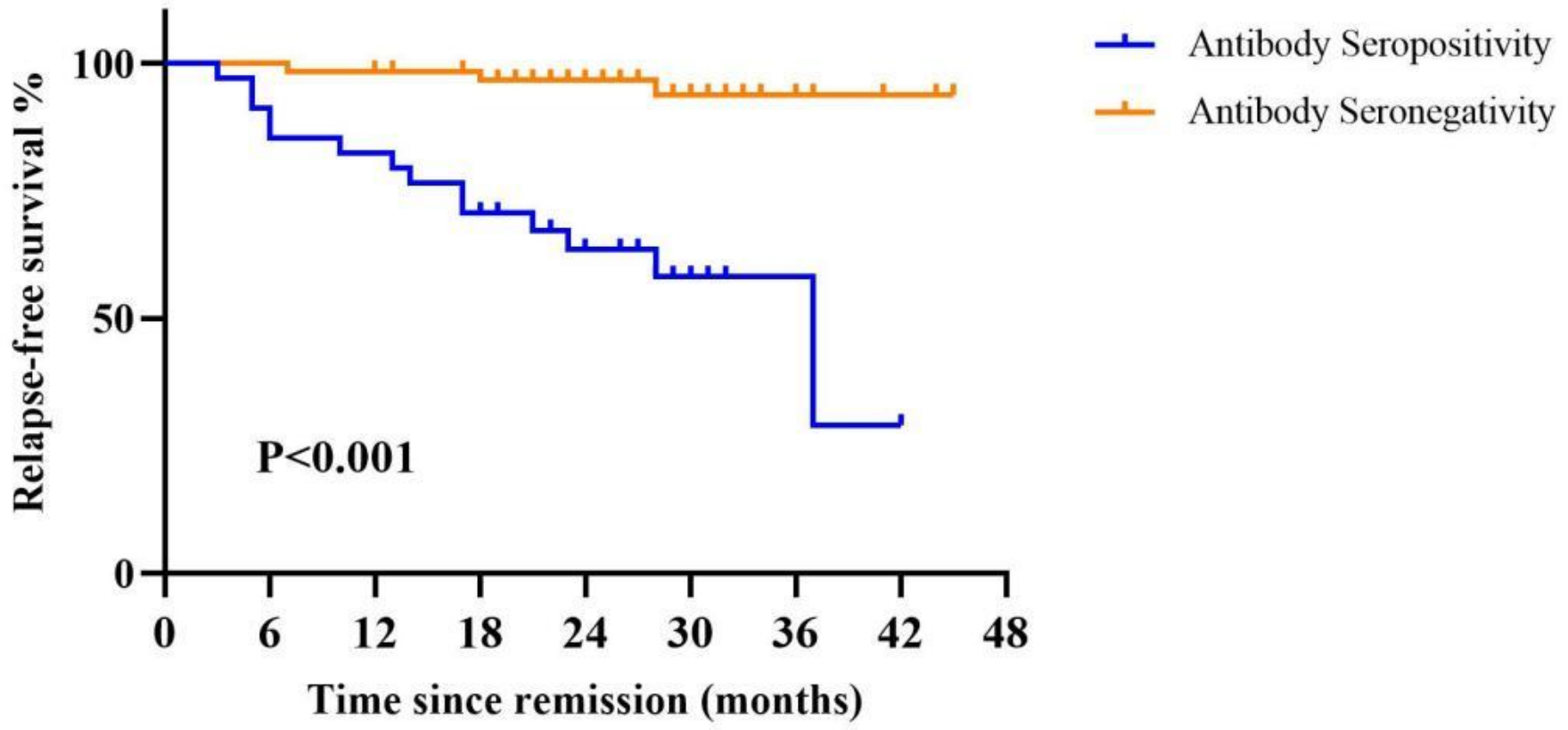

Number at risk									
Total	96	93	90	80	60	37	9	5	1
Anti-PLA2R Antibody Seropositivity	34	31	29	24	17	7	3	1	
Anti-PLA2R Antibody Seronegativity	62	62	61	56	43	30	6	4	1

Supplementary Figure 2. Kaplan-Meier curves for relapse-free survival from the date of remission, stratified by 3-month anti-PLA2R antibody status. Patients with 3-month anti-PLA2R antibody levels < 14 RU/mL were classified into the antibody seronegativity group, and those with levels ≥ 14 RU/mL into the antibody seropositivity group, using the manufacturer-specified seropositivity threshold. The log-rank test was used for comparison between groups.

Supplementary Table 1. Histological characteristics in pMN patients with remission at baseline

Histological Characteristics	Overall Cohort (n=96)	Relapse (n=17)	Non-relapse (n=79)	*P* value
Histological classification, n (%)				
Stage I	22(22.92)	6(35.29)	16(20.25)	
Stage II	60(62.50)	11(64.71)	49(62.03)	0.11
Stage III	14(14.58)	0	14(17.72)	
Interstitial fibrosis/tubular atrophy, n (%)				
<10%	93(96.88)	16(94.12)	77(97.46)	
10%-20%	2(2.08)	1(5.88)	1(1.27)	0.43
≥20%	1(1.04)	0	1(1.27)	
Global glomerulosclerosis, n (%)	44(45.83)	9(52.94)	35(44.30)	0.60
Segmental glomerulosclerosis, n (%)	21(21.87)	2(11.76)	19(24.05)	0.35

Supplementary Table 2. Clinical characteristics of the cohort at 6 months post-treatment

**Characteristics at 6 months**	Overall Cohort(n=96)	Relapse (n=17)	Non-relapse (n=79)	*P* value
Proteinuria at 6 mo, g/d	3.20(1.68-4.20)	3.25(1.81-4.61)	3.20(1.42-4.20)	0.62
Proteinuria change from baseline to 6 mo (decrease), g/d	4.38(3.48-5.49)	4.25(2.97-4.82)	4.44(3.53-5.84)	0.20
Albumin at 6 mo, g/L	36.70(34.90-38.90)	35.30(32.70-38.80)	36.85(35.47-39.05)	0.09
Albumin change from baseline to 6 mo (increase), g/L	10.40(8.50-14.00)	9.60(7.50-12.55)	11.40(8.57-14.00)	0.32
eGFR at 6 mo, ml/min per 1.73 m^2	100.55(92.22-109.85)	106.80(91.61-120.96)	99.20(92.20-108.80)	0.10
eGFR change from baseline to 6 mo (increase), ml/min per 1.73 m^2	1.55(-3.98-6.37)	1.80(-2.55-6.88)	1.30(-4.80-6.20)	0.65
PLA2R antibody level at 6 mo, RU/mL	5.00(5.00-10.00)	10.00(10.00-16.00)	5.00(5.00-10.00)	<0.001
PLA2R antibody levels at 6 mo, n (%)				
< 14 RU/mL	83(86.46)	10(58.82)	73(92.41)	
14-20 RU/mL	10(10.42)	6(35.30)	4(5.06)	<0.001
≥ 20 RU/mL	3(3.12)	1(5.88)	2(2.53)	
PLA2R antibody change from baseline to 6 mo (decrease), RU/mL	38.00(15.25-65.50)	60.00(44.50-89.50)	31.00(13.00-54.00)	0.002

P value < 0.05 were considered as significant. Abbreviations: eGFR, estimated glomerular filtration rate.

Supplementary Table 3. Univariable and multivariable Cox regression models for relapse using risk factors at baseline

Risk factors	Univariable Model		Multivariable Model	
	**HR (95% CI)**	***P* value**	**HR (95% CI)**	***P* value**
Anti-PLA2R antibody level, RU/mL	1.01(1.00-1.01)	0.08	1.01(1.00-1.01)	0.08
Proteinuria, g/d	0.89(0.72-1.10)	0.28	-	-
Albumin, g/L	0.97(0.87-1.08)	0.58	-	-
Treatment allocation (versus cyclophosphamide)				
RTX	0.46(0.09-2.38)	0.36	-	-
CNIs± GC	1.62(0.55-4.76)	0.38	-	-

HR, hazard ratio; CI, confidence interval; RTX, rituximab; CNIs, calcineurin inhibitors; GC, glucocorticoids.

Supplementary Table 4. Univariable and multivariable Cox regression models for relapse using risk factors at baseline and at 6 months

Risk factors	Univariable Model		Multivariable Model	
	HR (95% CI)	*P* value	HR (95% CI)	*P* value
PLA2R antibody change from baseline to 6 mo (per RU/mL, decrease)	1.00(1.00-1.01)	0.13	-	-
Anti-PLA2R antibody seropositivity at 6 mo	4.73(1.78-12.54)	0.002	4.76(1.80-12.61)	0.002
PLA2R antibody level at baseline (per RU/mL)	1.01(1.00-1.01)	0.08	-	-
Proteinuria change from baseline to 6 mo (per g/d, decrease)	0.73(0.55-0.98)	0.04	-	-
Proteinuria at baseline (per g/d)	0.89(0.72-1.10)	0.28	-	-
Albumin change from baseline to 6 mo (per g/L, increase)	0.97(0.91-1.04)	0.41	0.97(0.90-1.04)	0.39
Albumin at baseline (per g/L)	0.97(0.87-1.08)	0.58	-	-
Treatment allocation (versus cyclophosphamide)				
RTX	0.46(0.09-2.38)	0.36	-	-
CNIs± GC	1.62(0.55-4.76)	0.38	-	-

Anti-PLA2R antibody seropositivity at 6 months was defined as anti-PLA2R antibody levels ≥ 14 RU/mL. The reference group was patients with levels < 14 RU/mL (antibody seronegativity).

Supplementary Table 5. Treatment-specific sensitivity analyses of multivariable Cox regression models for relapse

Risk factors	RTX-excluded group (n=68)		CTX-excluded group(n=66)	
	HR (95% CI)	*P* value	HR (95% CI)	*P* value
Anti-PLA2R antibody seropositivity at 3 mo	6.15(1.72-22.02)	0.005	4.84(1.27-18.44)	0.02
Albumin change from baseline to 3 mo (per g/L, increase)	0.88(0.76-1.02)	0.08	0.84(0.72-0.98)	0.03

Anti-PLA2R antibody seropositivity at 3 months was defined as antibody levels ≥ 14 RU/mL (seropositivity) versus < 14 RU/mL (seronegativity). The RTX-excluded group comprised patients receiving CTX plus glucocorticoids or CNIs with or without glucocorticoids; the CTX-excluded group comprised patients receiving RTX or CNIs with or without glucocorticoids.

Supplementary Table 6. Model fit comparison at different landmark time points

Model	AIC	C-index
3 months		
Univariable: anti-PLA2R Ab seropositivity only	126.72	0.78
Multivariable: anti-PLA2R Ab seropositivity + Δ Albumin	123.41	0.83
6 months		
Univariable:anti-PLA2R Ab seropositivity only	137.48	0.67
Multivariable: anti-PLA2R Ab seropositivity + Δ Albumin	138.84	0.66

A lower AIC and higher C-index indicate better model fit. AIC, Akaike information criterion; C-index, Harrell's concordance index. Δ Albumin, change in serum albumin from baseline to the corresponding landmark time.
